# Reorganization of Anatomical Connectome following Electroconvulsive Therapy in Major Depressive Disorder

**DOI:** 10.1155/2015/271674

**Published:** 2015-12-06

**Authors:** Jinkun Zeng, Qinghua Luo, Lian Du, Wei Liao, Yongmei Li, Haixia Liu, Dan Liu, Yixiao Fu, Haitang Qiu, Xirong Li, Tian Qiu, Huaqing Meng

**Affiliations:** ^1^Department of Psychiatry, The First Affiliated Hospital of Chongqing Medical University, Chongqing 400016, China; ^2^Center for Cognition and Brain Disorders and the Affiliated Hospital, Hangzhou Normal University, Hangzhou 310015, China; ^3^Department of Radiology, The First Affiliated Hospital of Chongqing Medical University, Chongqing 400016, China; ^4^Medical Psychology Department, The Third Military Medical University, Chongqing 400016, China

## Abstract

*Objective*. Electroconvulsive therapy (ECT) is considered one of the most effective and fast-acting treatment options for depressive episodes. Little is known, however, about ECT's enabling brain (neuro)plasticity effects, particular for plasticity of white matter pathway. *Materials and Methods*. We collected longitudinal diffusion tensor imaging in the first-episode, drug-naïve major depressive disorder (MDD) patients (*n* = 24) before and after a predefined time window ECT treatment. We constructed large-scale anatomical networks derived from white matter fiber tractography and evaluated the topological reorganization using graph theoretical analysis. We also assessed the relationship between topological reorganization with improvements in depressive symptoms. *Results*. Our investigation revealed three main findings: (1) the small-worldness was persistent after ECT series; (2) anatomical connections changes were found in limbic structure, temporal and frontal lobes, in which the connection changes between amygdala and parahippocampus correlate with depressive symptom reduction; (3) significant nodal strength changes were found in right paralimbic network. *Conclusions*. ECT elicits neuroplastic processes associated with improvements in depressive symptoms that act to specific local ventral frontolimbic circuits, but not small-world property. Overall, ECT induced topological reorganization in large-scale brain structural network, opening up new avenues to better understand the mode of ECT action in MDD.

## 1. Introduction

Patients with major depressive disorder (MDD) typically experience persistent depressed/sad mood and are highly debilitating [1]. Although antidepressant medications and psychotherapeutic treatments are currently available in many patients, electroconvulsive therapy (ECT) is thought to be the most effective and fast-acting remission for depressive episodes [2, 3]. However, little is known about ECT's enabling brain (neuro)plasticity effects [4], particular for plasticity of white matter pathway [5].

Accumulated evidence suggests that the act of ECT on brain structure associated with clinical state and treatment response in MDD [4]. The hippocampal, amygdalar, and striatal subcortical centers have shown increases in gray matter volume after ECT series, suggesting ECT-induced brain structure neuroplasticity related to improved clinical response [6–9]. Furthermore, diffusion tensor imaging (DTI) can quantify the fiber orientation and integrity of white matter (WM) pathway within neural network. Early specific regions of interest- (ROI-) based DTI studies suggest a trend of increased WM microstructure (fractional anisotropy [FA]) in the hippocampal formation and frontal and temporal lobes following ECT treatment [10, 11]. Recently, whole-brain DTI work found increases of FA in dorsal frontolimbic circuits that are modulated by ECT therapy and relate to therapeutic response [12]. Taken together, MDD do not result from a deficit in a single brain region and local neuronal circuits which contributed to ECT-related structural plasticity. However, it is likely that a large-scale network perspective is necessary to explain their complex etiology and ECT's enabling brain neuroplasticity.

The human brain is structurally and functionally organized into connectome [13, 14]. Depression is also associated with abnormal topological organization of brain networks [15–17], which are valuable for diagnosis neuromarkers and treatment evaluation. In the current study, we collected longitudinal DTI in the first-episode, drug-naïve MDD patients before and after a predefined time window ECT treatment. We constructed large-scale anatomical networks derived from WM fiber tractography and evaluated the dynamic alteration of global, nodal topological characteristics and the strength of each connection using graph theoretical analysis. We aimed to directly answer whether neuroplasticity of ECT related to large-scale anatomical connectome. We also assessed the relationship between topological organization with depressive severity during the ECT series, to test above ECT-related network plasticity would accounts for improvements in depressive symptoms.

## 2. Materials and Methods

### 2.1. Participants

All examinations were carried out under the guidance of the Declaration of Helsinki 1975. The protocol was reviewed and approved by the Local Medical Ethics Committee of the First Affiliated Hospital of Chongqing Medical University. All these patients were first-episode MDD, without any treatment history before. The diagnosis of MDD was confirmed with a structured clinical interview for DSM-IV-TR Disorders (SCID-I/P, Chinese version) [18], along with scores ≥21 on the 24-item Hamilton Rating Scale for Depression (HAMD) [19]. According to the clinical guidelines of the Canadian Network for Mood and Anxiety Treatments, ECT could be considered as first-line treatment for depression with acute suicidal ideation, psychotic features, and catatonia [20]. Consequently, all the patients were required to have at least one of the above three characteristics. Patients were excluded if they (i) had any history of alcohol or drug abuse, neurological or serious physical disease, and morphological anomaly of the brain and (ii) had any surgical electronic or metal implants. MDD patients were scanned before one day of starting the ECT sessions. All the patients were scanned for the second time at least >1 day after eight ECT sessions. We did not control the medicine of subjects during this study, which was decided by their clinician independently. After the first ECT, most patients were given antidepressants over the course of ECT.

### 2.2. ECT Procedures

The patients underwent modified bitemporal ECT using a brief-pulse, constant current apparatus Thymatron (TM) DGx (Somatics LLC, Lake Bluff, IL, USA) at the Department of Psychiatry of the First Affiliated Hospital of Chongqing Medical University. The first three ECT administrations occurred on consecutive days, and the remaining ECT administrations were conducted every other day with a break of weekends until at least eight times of ECT [22]. After that, ECT treatments were continued if clinical depressive symptoms had not improved sufficiently, which were also decided by a clinician, but lasted at most 12 times of ECTs. The initial dosage selected was normalized, based on sex, age, and weight. Anesthesia was induced with propofol (1.5–2 mg/kg) and succinylcholine (0.5–1 mg/kg). We applied a stimulus dose up to 1.5–2 times above seizure threshold [20].

### 2.3. Clinical Assessments

The patients received scanning and depressive symptoms assessments at two separate time points: (i) within one day before their first ECT session (pre-ECT) and (ii) the day after completion of eight ECT sessions (post-ECT). Ratings of depressive symptoms were collected at each time point using the 24-item HAMD.

### 2.4. Image Acquisition and Preprocessing

We acquired imaging data using a 3.0 Tesla MRI system (GE Signa) in the First Affiliated Hospital of Chongqing Medical University. We used foam padding to minimize head motion. We acquired 3D T1-weighted anatomical images (repetition time = 8.35 ms, echo time = 3.27 ms, flip angle = 12°, field of view = 240 × 240 mm^2^, matrix = 256 × 256, slice thickness = 1 mm, and number of slices = 156 sagittal slices). We also acquired the diffusion-weighted images using spin echo-based echo planar imaging sequence (25 noncollinear directions, *b* = 1000 s/mm^2^, one volume without diffusion-weighted *b* = 0 s/mm^2^, number of slices = 37 axial slices, repetition time = 10000 ms, echo time = 86.7 ms, flip angle = 90°, field of view = 256 × 256 mm^2^, matrix = 256 × 256, slice thickness = 3 mm, and number of averages = 2).

DTI data were preprocessed and analyzed using the Pipeline for Analyzing Brain Diffusion Images toolkit (PANDA; http://www.nitrc.org/projects/panda) [24], which synthesizes procedures in FSL (http://fsl.fmrib.ox.ac.uk/fsl) and the diffusion toolkit. DTI data were coregistered to the B0 image and corrected for distortion induced by eddy currents. Diffusion tensor models were estimated by using the linear least-squares fitting method at each voxel by using the diffusion toolkit. Whole-brain MW fiber tracking was performed in the native diffusion space for each subject by using the fiber assignment with the continuous tracking algorithm. Path tracking proceeded until either the fractional anisotropy was less than 0.15 or the angle between the current and the previous path segment was greater than 35 degrees, as in our previous studies [25–27]. Fibers less than 10 mm or with obvious false paths were discarded.

### 2.5. Anatomical Connectome Construction

To determine the nodes of anatomical connectivity networks, we used the automated anatomical labelling (AAL) template [23] to parcellate the whole cerebral cortex into 90 noncerebellar anatomical ROIs. A list of anatomical labels of these ROIs are presented in Table 2. The ROIs were transformed into each subject's native diffusion space. Specifically, we coregistered the individual 3D-T1 images to B0 images and normalized the 3D T1 images to the Montreal Neurologic Institute space by a 12-parameter nonlinear transformation. These transformation parameters were inversed and applied to 90 ROIs.

In this native diffusion space, anatomical connectivity between ROI *i* and *j* was defined as the number of fibers connecting *i* and *j*. We selected fiber number as measurement because it is sensitive to the topological difference between patients with distinct outcomes. The raw network connectivity (fiber number) was scaled to the total volume of ROI *i* and *j* [25]. For each subject, the anatomical connectivity matrix “*M*” had 90 × 90 entries, with *A*_*ij*_ corresponding to the weighted connectivity between ROI *i* and *j*, also referred to as the link between nodes *i* and *j*.

### 2.6. Graph Theoretic Measures

Graph theory enables the quantification of network topological properties [28]. Graph measures for each individual connectivity matrix were calculated with the Brain Connectivity Toolbox (https://sites.google.com/site/bctnet/). Small-worldness is an optimal architecture balancing the segregation and integration of information, with similar path length (*λ* = *L*_net_^*w*^/*L*_random_^*w*^ ≈ 1) but higher clustering coefficient (*γ* = *C*_net_^*w*^/*C*_random_^*w*^ > 1) than a random network [29]. We evaluated the typical properties of a small-worldness (*σ* = *γ*/*λ*) that is typically larger than one. We selected nodal strength to estimate the topological feature of each node, because its high test-retest reliability [30] and clear neurophysiology relevance [31]. Nodal strength (*S*_*i*_) was computed as the sum of the weights of all the connections of node *i*. It quantifies the extent to which a node is relevant to the graph.

### 2.7. Statistical Analysis

To examine how anatomical architectures reorganized following ECT, we performed a paired *t*-test (post-ECT versus pre-ECT) for network measures. The anatomical connectivity edge, small-worldness, and nodal strength were analyzed, respectively. In addition, to explore associations between network measures changes (post-ECT − pre-ECT) and the depressive symptom reduction (pre-ECT − post-ECT HAMD scores) in patients after ECT (*n* = 22), we calculated the Pearson correlation coefficients. We also computed the relationship between the anatomical measures of the pre-ECT data and the duration of depressive episode. As these analyses were exploratory, we used an uncorrected statistical significance level of *P* < 0.05.

## 3. Results

### 3.1. Clinical Data

Twenty-four first-episode, drug-naïve MDD patients (15 female, all right-handed, age [mean ± SD]: 28.88 ± 10.77 years) who received eight ECT series have enrolled (Table 1). Note that two patients' clinical data were missed. Patients' HAMD score before ECT was 28.14 ± 5.43, indicating severe depression. After ECT series, depressive symptoms (HAMD score) significantly reduced (*T*_21_ = 11.92, *P* < 0.0001, paired *t*-test) (Table 1), indicating excellent therapeutic effects of ECT. Nineteen of the 22 patients (86.36%) were ECT responders whose depressive symptoms reduced at least to 50% compared to pre-ECT HAMD [32]. Ten of the 22 patients (45%) were in remission—that is, their HAMD scores were ≤ 7—after the ECT series. After the first ECT, 20 of the 22 patients (90.9%) were given antidepressants over the course of ECT.

### 3.2. ECT Effects on Topological Reorganization

The anatomical connectome exhibited typical features of small-word topology no matter pre- or post-ECT. However, no significant difference was found between post- and pre-ECT (*T* = 0.77, *P* = 0.447).

**Figure 1:**
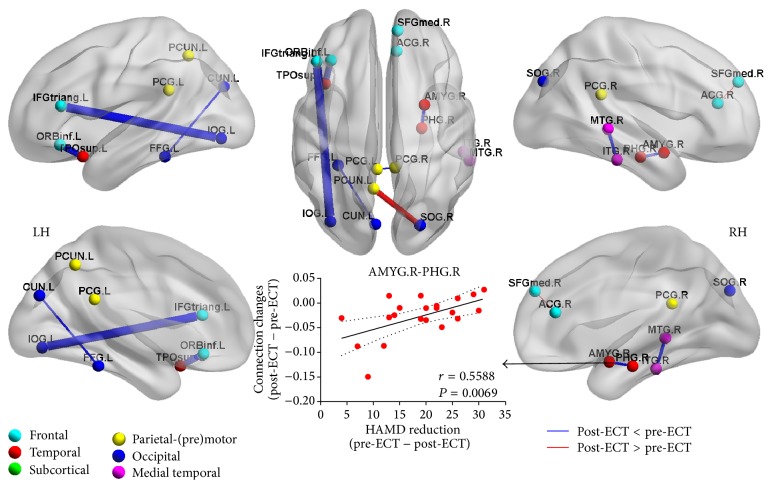
Significant differences in anatomical connection between post-ECT and pre-ECT. Nodes (individual ROIs) were differently colored according to the six anatomical modules listed in Table 2. Undirected edges were differently colored according to the significantly larger connection (*P* < 0.05). Nodes and edges are presented on inflated surface maps by BrainNet Viewer (http://www.nitrc.org/projects/bnv) [21]. Scatter-plot indicated the changed connection (post-ECT − pre-ECT) showing significant correlation with the HAMD reduction (pre-ECT − post-ECT).

Eight anatomical connections showed reorganization feature after ECT series (*P* < 0.01, corrected). The six increased connections were mainly located in limbic structure, temporal and frontal lobes, for example, amygdala (AMYG) versus parahippocampal gyrus (PHG), inferior temporal gyrus (ITG) versus middle temporal gyrus (MTG), and middle temporal pole (TPOsup) versus orbital inferior frontal gyrus (ORBinf), while the two connections, including anterior cingulate cortex (ACC) versus medial superior frontal gyrus (SFGmed) and precuneus (PCUN) versus superior occipital gyrus (SOG), showed decreased connection. In addition, the connection changes between amygdala and parahippocampus correlated with depressive symptom reduction (Figure 1).

**Figure 2:**
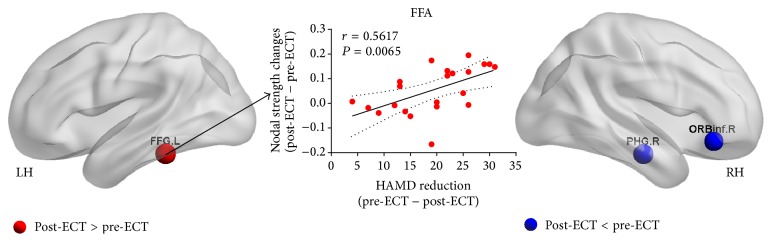
Significant differences in nodal strength between post-ECT and pre-ECT. Nodes (individual ROIs) were differently colored according to the significantly increased and decreased nodal strength (*P* < 0.05). Nodes are presented on inflated surface maps by BrainNet Viewer (http://www.nitrc.org/projects/bnv) [21]. Scatter-plot indicated the changed nodal strength in fusiform gyrus (post-ECT − pre-ECT) showing significant correlation with the HAMD reduction (pre-ECT − post-ECT).

A comparison of post-ECT treatment and pre-ECT treatment on nodal strength revealed significant increases in right PHG (*T* = 2.207, *P* = 0.037) and ORBinf (*T* = 2.073, *P* = 0.049) and decreases in left fusiform gyrus (FFG) (*T* = −2.285, *P* = 0.031) (Figure 2). The nodal strength changes of FFG correlated with depressive symptom reduction (Figure 2).

There is no significant correlation between the anatomical measures of the pre-ECT data and the duration of depressive episode.

## 4. Discussion

We investigated the reorganization mechanism of anatomical connectome, rather than local brain structure alone, in first-episode, drug-naïve MDD patients subsequent to ECT treatments. Our investigation revealed three main findings: (1) the small-worldness was persistent after ECT series; (2) significant anatomical connections changes were found in limbic structure, temporal and frontal lobes, in which the connection changes between amygdala and parahippocampus correlated with depressive symptom reduction; and (3) significant nodal strength changes were found in right paralimbic network. These findings would support the that ECT-induced brain structure neuroplasticity relates to improved clinical response [9].

The human brain is a complex network organized with a small-world property (high efficiency at a low wiring cost) [33]. This economic architecture was altered in the patients, suggesting a disturbance of the normal balance of segregation and integration of information in anatomical connectome in depression [15, 17]. For the first time, we revealed whether the small-world property is adaptive following the ECT series. Not surprising, the small-worldness was persistent, given that the small-world topology reflects an optimal balance between global integration and local special [34], without evidence for ECT-related change in global topology. Furthermore, the functional connectome is thought to be more flexible, while the anatomical connectome is relatively stable [25, 35]. We therefore reasoned that the anatomical connectivity network may be less affected by ECT in MDD.

Although the above-mentioned global topology was persistent, the anatomical connections showed reorganization in ventral and dorsal frontolimbic structure following ECT series. Previous cross-sectional MDD studies have consistently shown that increased anatomical connections were mainly located in cortical-limbic network, particularly in the frontolimbic network [15, 36, 37]. These prior findings support a dysregulation between underactive dorsal and overactive ventral frontolimbic circuitry [12]. Though few DTI studies have assessed treatment effects in MDD patients, our longitudinal results was compatible with prior findings. Our results further demonstrate ECT-related changes in the anatomical connections of selected frontolimbic pathways.

The amygdala and hippocampus play a pivotal role in regulation of emotion and in responses to emotion [38, 39]. The effects of ECT on amygdala and hippocampus volume suggest that ECT-induced brain gray matter structure neuroplasticity relates to improved clinical response [6–9]. We considered the anatomical basis connecting them, rather than alone, because the hippocampus connects with the amygdala and the limbic hypothalamic-pituitary-adrenal (HPA) axis, which are central to the pathophysiology of the MDD [9, 40]. In the current work, the fiber number was used to index the anatomical connection, which are closely related to neurite components of synapses (synaptogenesis) in neuroplasticity model [4]. Decreased connection would reflect axonal pruning [41], which regulate the HPA system activity [42]. Furthermore, these connection changes associate with HMDA reduction, suggesting that ECT-related anatomical connection plasticity may contribute to improvements in depressive symptoms.

The post-ECT and pre-ECT comparison of nodal topological characteristic, for example, nodal strength, revealed alterations or neuroplasticity of network hubs in MDD patients. The characteristic of nodal strength is the most fundamental network measure with high reliability [30], neurophysiology relevance [31], and sensitive neuromarkers of MDD [16]. The changes of nodal strength in right hippocampus and inferior frontal lobe, consistent with prior local brain plasticity in ventral frontolimbic structure. Rather unexpectedly, we also observed that the fusiform gyrus presented increases nodal topological characteristics following ECT series. This change was furthermore shown to be positively correlated with depressive symptoms relevance. A more recent meta-analysis in MDD patients pointed to smaller FA in fusiform gyrus that is involved in inferior longitudinal fasciculus [43]. The role of the fusiform gyrus in memory processing and face recognition may contribute to cognitive vulnerability to depression [44]. Our finding of increased nodal strength under this gyrus may provide structural evidence of white matter plasticity in the neural circuit in MDD from a connectome point of view.

Several methodological limitations need to be addressed. First, in absence of a healthy control group, we would not assess the normalization of aberrant network measures when compared post-ECT depression group and controls [45] and predict the treatment response/remission when compared pre-ECT depression group and controls [46]. Second, the patients were only scanned twice within a predefined time window. More scans would conduce to explore the relation between times of ECT and neuroimaging changes for each patient [47]. In addition, although ECT showed antidepressant therapeutic effect, the ECT-related cognitive side effects have not been considered here, which are worthy of concern in future studies [48]. Finally, although diffusion tensor modal is an effective way to investigate white matter networks, some advanced approaches (e.g., diffusion spectrum imaging) may lead to more accurate tractography results, particularly in areas with cross fibers.

## 5. Conclusion

ECT elicits neuroplastic processes associated with improvements in depressive symptoms that act to specific local ventral frontolimbic circuits, but not global small-world property. ECT-related changes in the anatomical connections of selected amygdala and hippocampus associate with axonal pruning, which regulate the HPA system activity. Our finding of increased nodal strength under fusiform gyrus may provide structural evidence of white matter plasticity in the neural circuit in MDD from a connectome point of view for adapting cognitive vulnerability. Overall, ECT induced topological reorganization in large-scale brain structural network, opening up new avenues to better understand the mode of ECT action in MDD.

## Figures and Tables

**Table 1:** Demographic and clinical characteristics of patients.

Demographics	MDD (*n* = 24)		
Age (years)	28.88 ± 10.77		
Sex (male/female)	9/15		
Education (years)	11.96 ± 2.79		
Age of onset (years)^*∗*^	26.62 ± 12.12		
Suicidal thought or behavior (%)^*∗*^	77.27		
Duration of depressive episode (months)^*∗*^	2.83 ± 5.43		

	Pre-ECT	Post-ECT	*P* value

HAMD^*∗*^	28.14 ± 5.43	8.81 ± 4.20	<0.0001^a^

MDD, major depressive disorder; HAMD, Hamilton Rating Scale for Depression; ECT, electroconvulsive therapy.

The values are illustrated as mean ± SD.

^*∗*^Two patients' clinical data missed.

^a^Paired *t*-test.

**Table 2:** Regions of interest (ROI) in the AAL template.

Region name	Abbreviation
*Medial temporal *	
Amygdala	AMYG
Hippocampus	HIP
Parahippocampal gyrus	PHG
Middle temporal gyrus, temporal pole	TPOsup
Superior temporal gyrus, temporal pole	TPOmid
*Subcortical *	
Caudate nucleus	CAU
Olfactory cortex	OLF
Palladium	PAL
Putamen	PUT
Thalamus	THA
*Occipital *	
Calcarine fissure	CAL
Cuneus	CUN
Fusiform gyrus	FFG
Lingual gyrus	LING
Inferior occipital gyrus	IOG
Middle occipital gyrus	MOG
Superior occipital gyrus	SOG
*Temporal *	
Heschl gyrus	HES
Insula	INS
Inferior temporal gyrus	ITG
Middle temporal gyrus	MTG
Superior temporal gyrus	STG
*Frontal *	
Anterior cingulate cortex	ACC
Inferior frontal gyrus, opercular	IFGoper
Inferior frontal gyrus, orbital	ORBinf
Inferior frontal gyrus, triangular	IFGtri
Superior frontal gyrus, medial orbital	SFGmorb
Middle frontal gyrus, orbital	MFGorb
Middle frontal gyrus	MFG
Superior frontal gyrus, medial	SFGmed
Superior frontal gyrus, orbital	SFGorb
Superior frontal gyrus	SFG
Gyrus rectus	REG
*Parietal-(pre) Motor *	
Rolandic operculum	ROL
Angular gyrus	ANG
Median cingulate gyrus	MCC
Posterior cingulate gyrus	PCC
Paracentral lobule	PCL
Inferior parietal gyrus	IPG
Superior parietal gyrus	SPG
Postcentral gyrus	PoCG
Precentral gyrus	PreCG
Precuneus	PCUN
Supplementary motor area	SMA
Supramarginal gyrus	SMG

The abbreviations used in the study differ slightly from the original abbreviations by Tzourio-Mazoyer et al. [23].
